# Buformin suppresses osteosarcoma via targeting AMPK signaling pathway

**DOI:** 10.1515/biol-2020-0041

**Published:** 2020-06-30

**Authors:** Yan Ding, Shiqiao Lv, Guangrun Li, Jinpeng Cui, Yunzhen Chen

**Affiliations:** Department of Spine, Qilu Hospital, Cheeloo College of Medicine, Shandong University, No. 107 West Wenhua Road, Jinan 250012, Shandong Province, China; Department of Orthopedics, Yantaishan Hospital, Yantai 264000, China; Department of Spine, Yantai Yuhuangding Hospital Affiliated to Medical College of Qingdao University, Yantai 264000, China; Clinical Laboratory, Yantaishan Hospital, Yantai 264000, China

**Keywords:** buformin, osteosarcoma, AMPK signal pathway, synergistic effect

## Abstract

**Background:**

Buformin has been reported to be a powerful anticancer drug by activating the AMPK signal. Herein, we aimed to investigate the effects of buformin on osteosarcoma.

**Material and methods:**

Cellular proliferative abilities were determined by cell counting kit-8 and colony formation assays. Cellular invasion was investigated using a transwell system. Cell cycle was examined by flow cytometry. Western blot was performed to measure the expression of key proteins. Synergistic effects of buformin and cisplatin were validated in seven fresh osteosarcoma tissues.

**Results:**

Buformin suppressed the growth of U-2 OS cells in a dose-dependent manner (IC50 = 69.1 µM). Moreover, buformin induced cell cycle arrest (*P* < 0.001) and impaired cellular invasion (*P* = 0.038). Phosphorylation of AMPK was upregulated by buformin, while phosphorylation of S6, cyclin D1, and MMP9 were significantly downregulated. In addition, buformin notably induced accumulation of reactive oxygen species and lactate and eventually decreased ATP production. In both U-2 OS cells and the primary cultured osteosarcoma tissues, buformin increased tumor sensitivity to cisplatin.

**Conclusions:**

Buformin could suppress tumor growth and invasion of osteosarcoma through directly targeting the AMPK signaling pathway. Moreover, buformin inhibited the abnormal metabolism and notably increased the cytotoxicity of cisplatin, and therefore represents a new potential treatment option for osteosarcoma.

## 1 Introduction

In children and adolescents, osteosarcoma is the most common malignancy originating from bone [1]. Globally, the incidence of osteosarcoma is approximately 3.4 cases per million people every year [2]. Histologically, the characteristics of osteosarcoma include the presence of malignant mesenchymal cells and the production of bone stroma, which shows a high tendency of lung metastasis. Currently, the combination of surgery and chemotherapy (cisplatin, paclitaxel, ifosfamide, and doxorubicin) is still the first choice to treat osteosarcoma [3]. Unfortunately, although several improvements have been achieved during the past few decades, the treatment of osteosarcoma remains a huge challenge. It has been reported that more than 50% of osteosarcoma patients do not benefit much from the current therapy [4,5,6]. Therefore, novel drugs and therapeutic approaches are urgently needed.

In the past twentieth century, biguanides, especially metformin, were widely prescribed for patients with type 2 diabetes mellitus [7]. The main mechanism is that biguanides could activate the AMPK signal and block mTOR and IGFR/IGF pathway, thereby enhancing cellular sensitivity to insulin. Moreover, biguanides were found to be able to suppress cancer proliferation, invasion, angiogenesis, and metabolism, both *in vitro* and *in vivo* [8,9,10,11,12]. Consistently, epidemiologic findings supported the notion that the long-term use of biguanides notably reduces the risk and improves the survival of cancer patients [13,14,15,16].

Although it is more powerful than metformin and phenformin, buformin was ejected from most markets because the incidence of acidosis was higher [17,18]. Recently, several studies have proposed that buformin could be used to prevent and treat various cancers, either alone or combined with other drugs or radiotherapy. In mouse model, buformin significantly reduced the risk of breast carcinoma [19,20]. Moreover, in endometrial cancer and cervical cancer, *in vitro* and *in vivo* results showed that buformin could suppress tumor growth and invasion through activating the AMPK signal and regulating metabolism [21,22]. Herein, we aim to investigate the functions of buformin in treating osteosarcoma.

## 2 Materials and methods

### 2.1 Sample collection and primary culture

For the primary culture, seven fresh tumor samples were obtained during surgery from Yantaishan Hospital. In brief, fresh tumor tissues were rapidly cut into pieces and digested in collagenase IA solution (C-9891; Sigma, USA; the concentration is 0.2%) at 37°C for 3–6 h. Then the tumor cells were collected by a short centrifugation and plated in a 96-well plate for further assays.


**Informed consent:** Informed consent has been obtained from all individuals included in this study.
**Ethical approval:** The research related to human use has been complied with all the relevant national regulations, institutional policies and in accordance with the tenets of the Helsinki Declaration and has been approved by the Ethics Committee of Yantai Yuhuangding Hospital Affiliated to Medical College of Qingdao University.

### 2.2 Cell lines and reagents

The human osteosarcoma U-2 OS cell line was provided by Dr Dongsheng Pei (Xuzhou Medical College, Jiangsu, China). Cells were routinely cultured in Dulbecco’s Modified Eagle Medium (DMEM) supplemented with 10% fetal bovine serum.

Buformin was purchased from Sigma (SML1496), and cisplatin was purchased from Shanghai Abmole Bioscience (M2223, China). The other reagents (if not specially mentioned) were all obtained from Beyotime (Shanghai, China).

### 2.3 CCK-8 assay

The suppressive effects of buformin on U-2 OS cells were measured using a CCK-8 (cell counting kit-8, M4839, Abmole Bioscience) assay. In brief, osteosarcoma cells were plated in 96-well plates (3 × 10^3^ cells/well) and cultured overnight. Then different doses of buformin were added to the medium. At the indicated time points, 10 µL of CCK-8 solution was added into each well and incubated for 1 h. The absorption (wavelength = 490 nm) was measured on a microplate reader.

### 2.4 Colony formation assay

One hundred U-2 OS cells were plated in a 10 cm dish and cultured overnight at 37°C. Then these cells were treated with 50 µM buformin for 2 weeks. The medium (containing buformin) was replaced every 5 days. On the fourteenth day, the dish was rinsed three times with phosphate-buffered saline (PBS) and then 1 mL of crystal violet solution was used to stain the attached cells. The colony (≥50 U-2 OS cells) number was determined on an inverted microscope.

### 2.5 Measurement of reactive oxygen species (ROS)

The production of ROS was measured using a commercial ROS kit purchased from Beyotime (S0033). In brief, 5 × 10^3^ cells/well were plated in black 96-well plates and incubated overnight at 37°C. After a 6-h treatment with buformin, 10 µM 2,7-Dichlorodi-hydrofluorescein diacetate was added into each well and reacted for 20 min. Then the fluorescence in each well (excitation = 488 nm, emission = 525 nm) was measured using a plate reader. All these procedures were performed in triplicate.

### 2.6 Cell cycle analysis

The cell cycle of osteosarcoma cells was examined using a FACSCalibur system. Cell culture and buformin treatment procedures were the same as described above. The cells were stained with propidium iodide, and the cell cycle distribution was determined on the FACSCalibur system.

### 2.7 Western blotting

Osteosarcoma cells or tissues were lysed in RIPA solution (Beyotime) for protein extraction. Proteins were separated by SDS–PAGE (with equal amounts of protein samples in each lane) and transferred to a PVDF membrane. The membranes were incubated with specific primary antibodies (anti-AMPK: AF1627, 1:500; anti-p-AMPK: AA393, 1:500; anti-S6: AF7917, 1:500; anti-p-S6: AF5917, 1:500; anti-cyclin D1: AF1183, 1:1,000; anti-MMP9: AF5234, 1:1,000) overnight at 4°C. On the second day, membranes were rinsed with phosphate buffered solution with Tween-20 and incubated with appropriate second antibodies for 2 h at room temperature. The specific protein bands were visualized using an ECL chemiluminescence kit (P0018; Beyotime).

### 2.8 Invasion assay

The invasive ability of osteosarcoma cells was examined using a transwell system (Beyotime). Briefly, 1 × 10^5^ osteosarcoma cells were plated in the upper chamber coated with Matrigel and 400 µL of DMEM containing 50 µM buformin was added to each well 6 h later. After a 24-h treatment with buformin, the upper chamber was rinsed with PBS and U-2 OS cells attached to the bottom were fixed with chilled methanol and stained with crystal violet solution. The cells that had invaded through the membrane were counted under an inverted microscope.

### 2.9 Measurement of ATP production

The alterations in ATP production were measured using an ATP assay kit purchased from Beyotime (S0026). In brief, 5 × 10^3^ cells/well were cultured in the 96-well plates and treated with different doses of buformin (ranging from 0 to 200 µM). Twenty-four hours later, 100 µL of detection solutions was added to each well and the luminescence intensity was measured on an illuminometer.

### 2.10 Measurement of lactate production

The production of lactate was tested by AAT Bioquest kit (13815, CA, USA). After the treatment with buformin, the medium in each group was collected at indicated time points, and 10 µL of medium was mixed with 90 µL of lactate detection solution. The mixture was placed in a 37°C water bath for 30 min, and then the absorbance was measured on a plate reader.

### 2.11 LDH activity assay

The activity of lactic dehydrogenase (LDH) was determined using a commercial kit of Beyotime (C0016, LDH activity assay kit). All of the procedures were performed according to the protocol, and the absorbance (wavelength = 490) was measured on a plate reader.

### 2.12 Statistical analysis

Statistical analysis was performed using the SPSS 22.0 software. Data were expressed as mean ± SE. Student’s *t*-test or one-way analysis of variance (ANOVA) was used for quantitative or categorical data, respectively. *P* values less than 0.05 indicate statistical significance.

## 3 Results

### 3.1 Buformin suppressed proliferation of osteosarcoma cells via activating the AMPK signaling pathway

**Figure 1:**
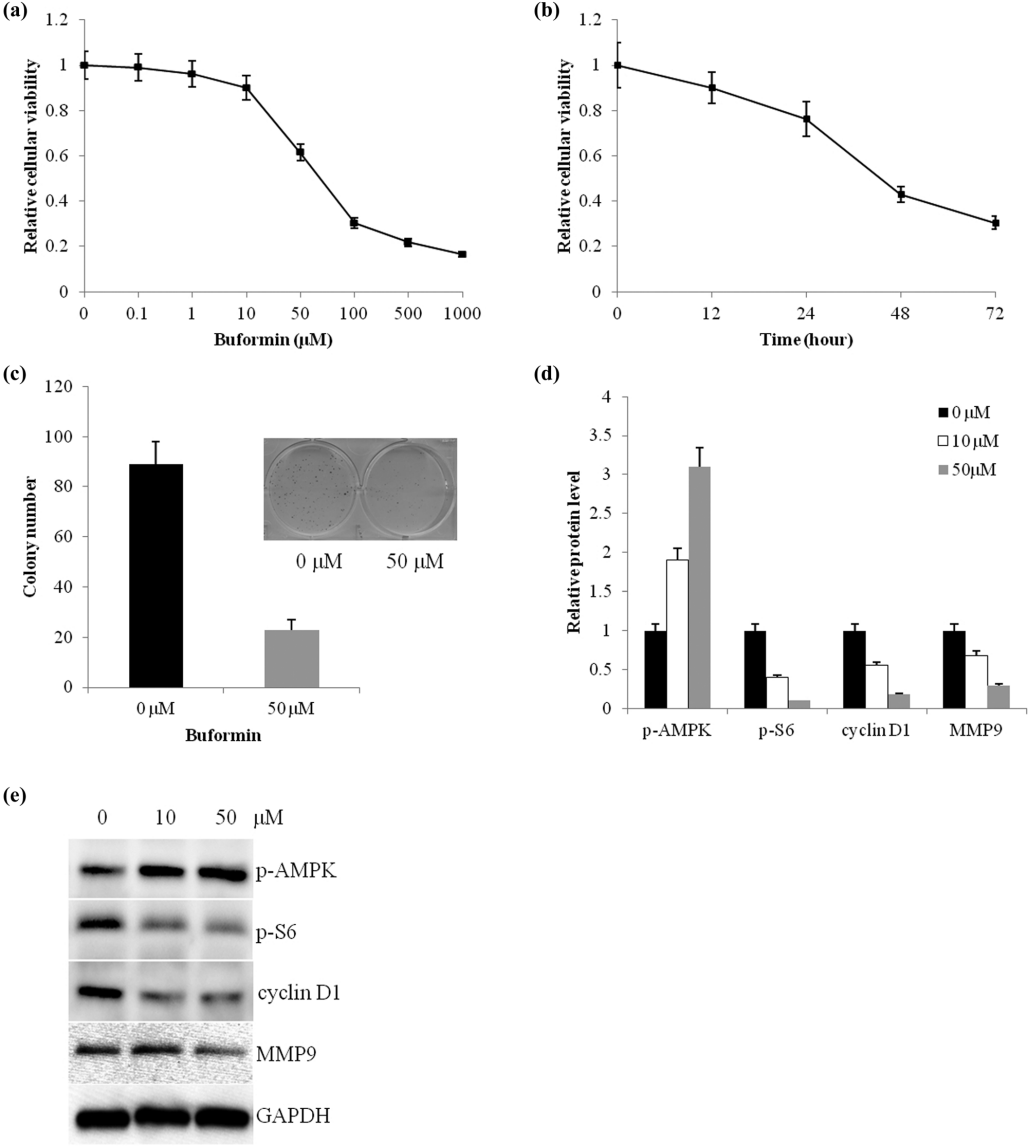
Buformin suppresses the proliferation of osteosarcoma cells via activating the AMPK signaling pathway. (a) The 72-h treatment with buformin significantly suppressed the proliferation of U-2 OS cells in a dose-dependent manner (IC50 = 69.1 µM, *P* = 0.017, one-way ANOVA was used for the statistical analysis); (b) the suppressive effects of buformin also are in a time-dependent manner (one-way ANOVA was used for the statistical analysis); (c) a 2-week treatment with 50 µM buformin sharply decreased the clones formed by U-2 OS cells (*P* = 0.016, Student’s *t*-test was used for the statistical analysis); (d and e) buformin activated the AMPK signal pathway and inhibited the activity of S6, cyclin D1, and MMP9.

According to the results of the CCK-8 assay, buformin exerted a significant dose-dependent suppressive effect on the growth of U-2 OS cells (Figure 1a, IC50 = 69.1 µM, *P* = 0.017, treated with buformin for 72 h). Moreover, we then repeated this assay using 100 µM buformin and found that buformin exerted suppressive effects in a time-dependent manner (Figure 1b).

In the colony formation assay, a 2-week treatment with 50 µM buformin notably decreased the number of osteosarcoma cell colonies (*P* = 0.016, Figure 1c). Considering that the AMPK is a well-identified target of buformin, we then tested the alterations of key factors involved in the AMPK signaling pathway. Our results proved that buformin significantly activated AMPK by upregulating its phosphorylation. Consequently, the phosphorylation of S6 was suppressed and the expression of cyclin D1 and MMP9 was downregulated (Figure 1d and e). These results illustrated the suppressive functions of buformin on proliferation and invasion.

### 3.2 Buformin arrested cell cycle progression and inhibited invasion in U-2 OS cells

**Figure 2:**
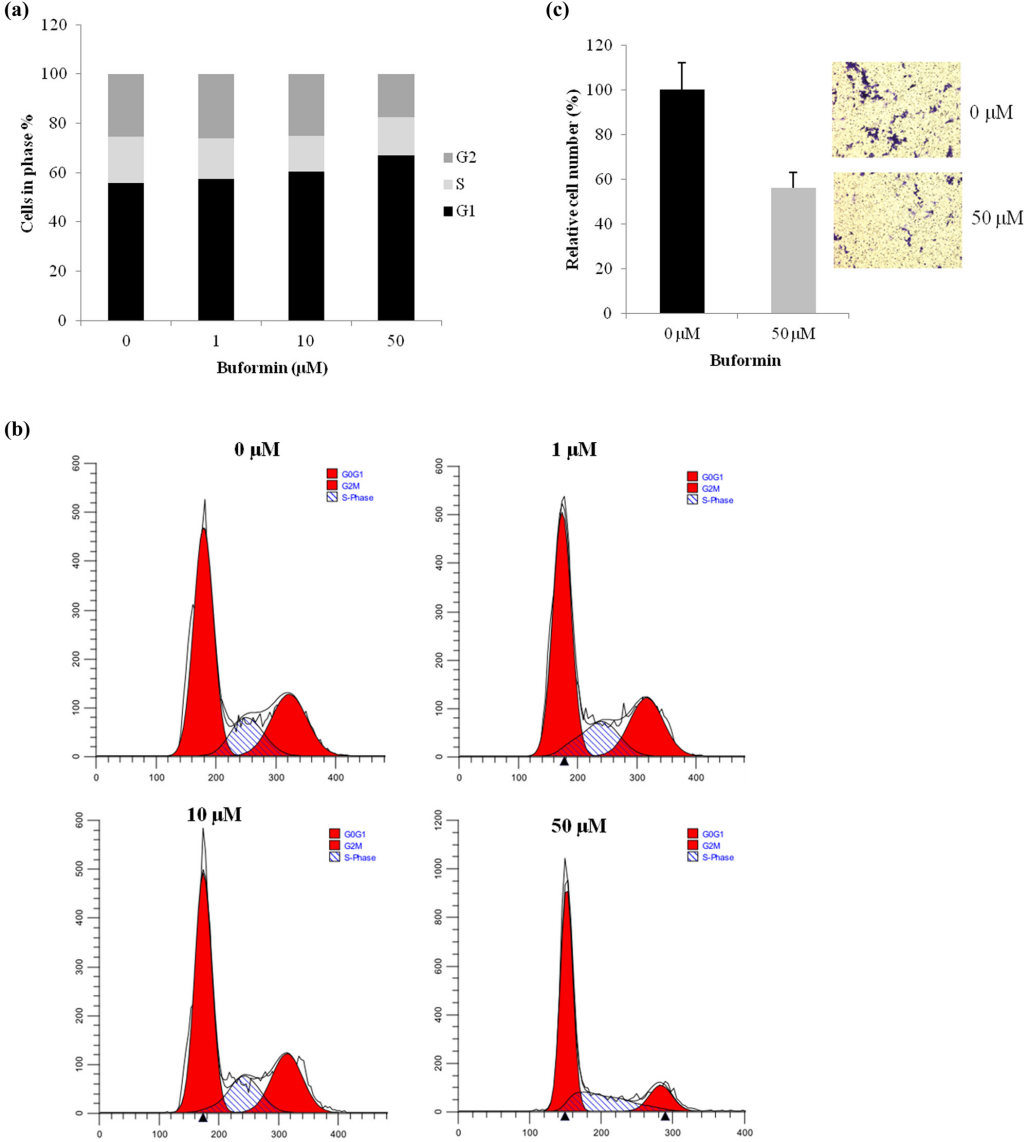
Buformin arrests cell cycle and inhibits invasion in U-2 OS cells. (a and b) Buformin led to a notable G1-phase arrest (*P* < 0.001, one-way ANOVA was used for the statistical analysis); (c) compared to the control, buformin significantly impaired the invasiveness of U-2 OS cells by inhibiting cells to penetrate through Matrigel (*P* = 0.038, Student’s *t*-test was used for the statistical analysis).

Next, we investigated the effects of buformin on cell cycle kinetics using the assay of flow cytometry. Compared to the control, a significant G1-phase arrest was detected in U-2 OS cells treated with 50 µM buformin (Figure 2a and b, *P* < 0.001). This finding was in consistent with the downregulation of cyclin D1. However, the 24-h treatment with buformin had no significant influence on cellular apoptosis (data not shown). Moreover, we examined the invasive ability of U-2 OS cells with a transwell system. Compared to the normal control, we found that the cellular invasive ability was decreased by more than 40% in the buformin group (*P* = 0.038, Figure 2c). These findings are also consistent with the downregulation of MMP9.

### 3.3 Buformin induced accumulation of ROS and restricted energy production in osteosarcoma cells

**Figure 3:**
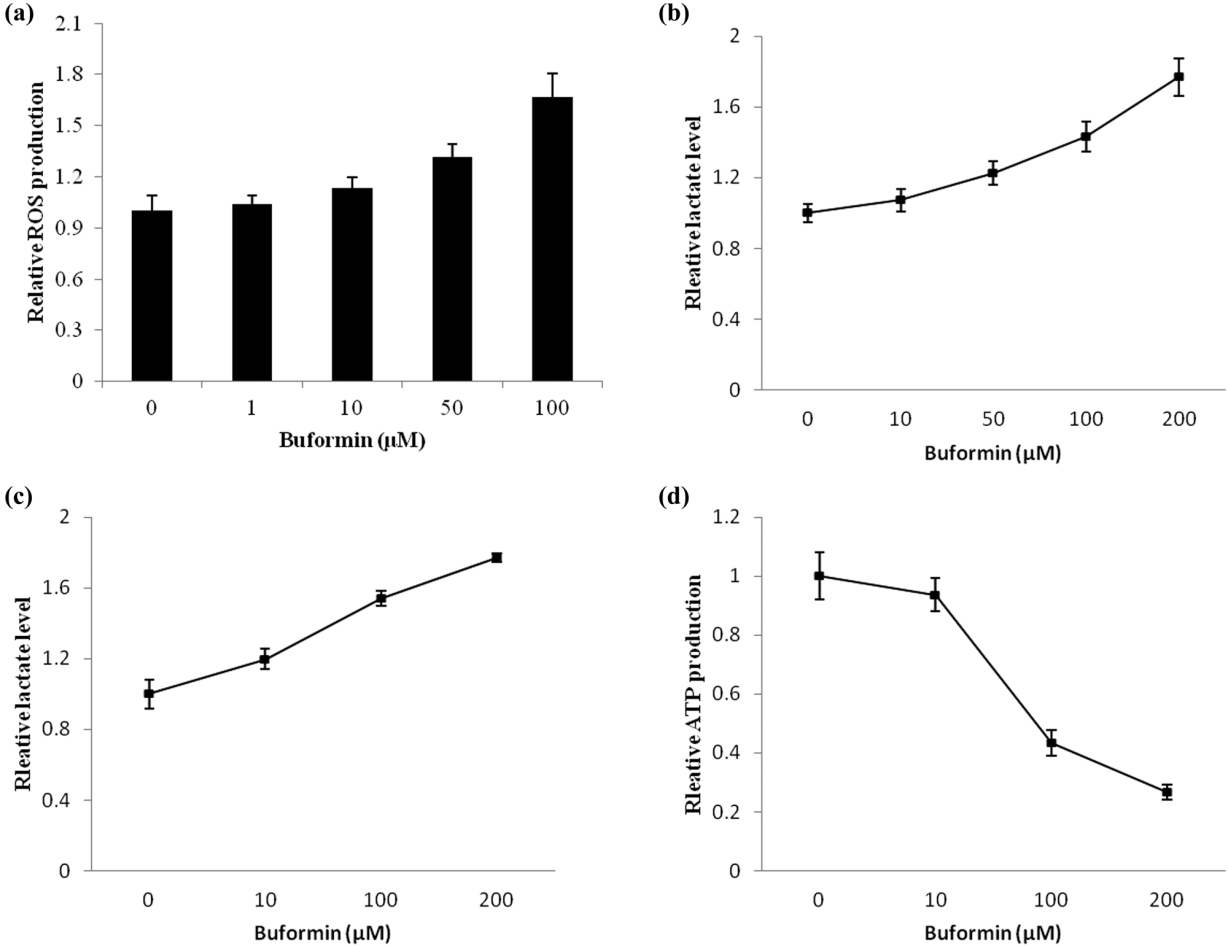
Buformin induces ROS and restricts energy production in osteosarcoma cells. (a) The production of ROS was significantly upregulated by a 6-h treatment of buformin in U-2 OS cells (*P* = 0.011, one-way ANOVA was used for the statistical analysis); (b–d) buformin significantly promoted the production of lactate (*P* = 0.029) by increasing LDH activity (*P* = 0.017), which consequentially led to a sharp decline in ATP level (*P* < 0.001, one-way ANOVA was used for the statistical analysis).

ROS is a sensitive marker of drug-induced cellular stress, which could be easily detected using commercial kits. We found that the production of ROS was significantly upregulated by buformin in U-2 OS cells (*P* = 0.011, Figure 3a).

Considering glycolysis is the main source of energy in osteosarcoma cells (the Warburg effect), we then tested how buformin affects metabolism in U-2 OS cells. As shown in Figure 3b and c, the 24-h treatment with buformin significantly increased the production of lactate (*P* = 0.029) by increasing LDH activity (*P* = 0.017), which consequentially led to a sharp decline in ATP levels (*P* < 0.001, Figure 3d). Collectively, our findings supported the notion that buformin can significantly restrict the energy supply of osteosarcoma cells, which was critical for its anticancer functions.

### 3.4 Buformin synergized with cisplatin in U-2 OS cells and primary cultured osteosarcoma tissues

**Figure 4:**
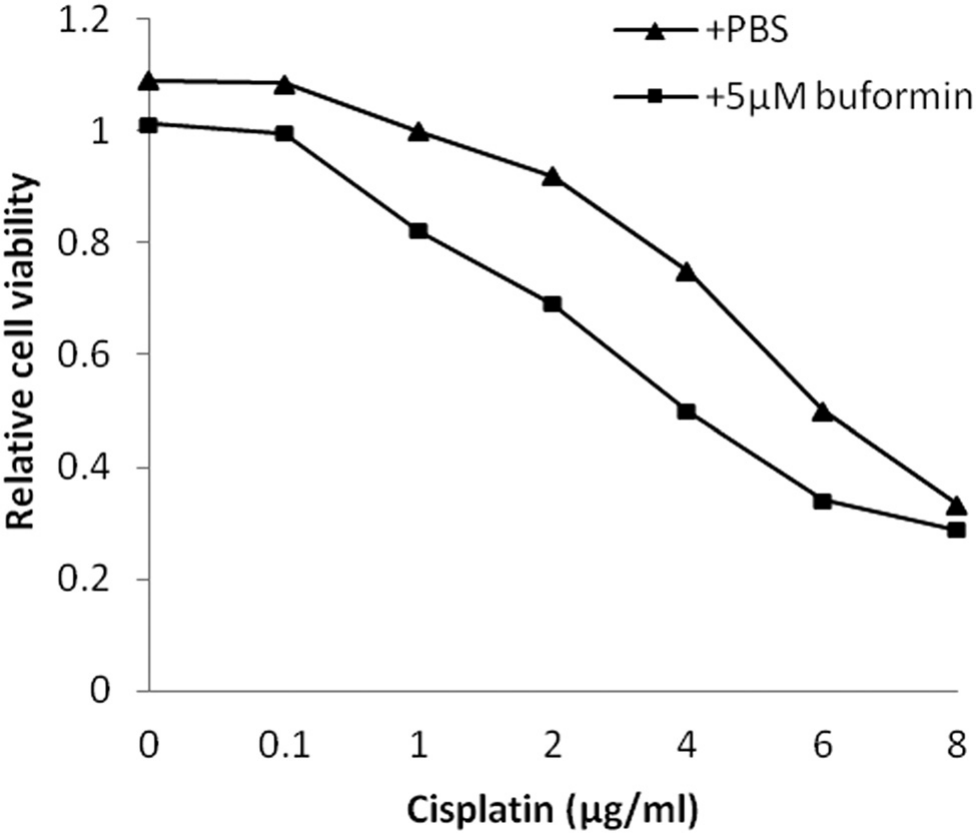
Synergistic effect of buformin and cisplatin in U-2 OS cells.

Since biguanides have been found to be chemotherapy sensitizers, we evaluated the mutual effects of buformin and cisplatin (a first-line drug for osteosarcoma). As our data show, a low-dose (5 µM) buformin is a suitable concentration for the synergy investigation, as it could achieve about 15% suppressive rate but would not cover the cytotoxicity of cisplatin. As shown in Figure 4, the treatment with 5 µM buformin significantly enhanced the cytotoxicity of cisplatin (ranging from 0.1 to 6 µg/mL). Confidence interval (CI) values (calculated according to the Chou–Talalay equation) also indicated a synergistic effect of buformin and cisplatin (Table 1; CI < 1: synergistic effect, CI = 1: additive effect, CI > 1: antagonistic effect).

**Table 1:** Effect of 5 µM buformin on cellular sensitivity to cisplatin in U-2 OS cells.

Buformin	Cisplatin	CI values
5 µM	0.1 µg/mL	0.991
5 µM	1 µg/mL	0.930
5 µM	2 µg/mL	0.552
5 µM	4 µg/mL	0.355
5 µM	6 µg/mL	0.469
5 µM	8 µg/mL	0.999

CI < 1: synergistic effect; CI = 1: additive effect; CI > 1: antagonistic effect.

To further validate this synergy, we primarily cultured seven fresh osteosarcoma tissues for the CCK-8 assay. As shown in Table 2, the addition of 5 µM buformin notably improved the cellular sensitivity to cisplatin in six of the seven (85.7%) samples, confirming the synergy between buformin and cisplatin.

**Table 2:** Interaction between buformin and cisplatin in primary cultured osteosarcoma tissues.

						IC50 to cisplatin (µg/mL)	
Case	Age	Gender	Stage	Subtype	Differation	+PBS	+5 µM Buformin
1	13	Female	IIb	Osteoblastic	Poor	9.95	6.69*
2	19	Female	IIa	Chondroblastic	Poor	15.36	1.97**
3	25	Male	Ib	Osteoblastic	Intermediate	7.55	4.31*
4	11	Male	III	Fibroblastic	Poor	11.61	3.95**
5	36	Female	IIa	Osteoblastic	Poor	18.85	17.59
6	9	Male	IIb	Osteoblastic	Poor	6.66	3.96**
7	20	Male	IIa	Chondroblastic	Poor	10.70	7.82*

## 4 Discussion

Currently, there are few effective treatments for osteosarcoma due to its rapid progression and poor responses to radio-/chemotherapy [23]. Most importantly, many patients are children and adolescents, so even if they can achieve long-term survival, radio-/chemotherapy could lead to much harm to their future lives [24,25]. Therefore, the development of novel, less harmful approaches is an urgent issue.

Abnormal metabolism, which provides essential energy and substances for proliferation, is a hallmark of cancer [26]. Thus, targeting the key factors manipulating cancer metabolism could be a potent strategy, especially for tumors characterized by rapid growth [27,28]. During the past few decades, biguanides, including buformin, have been demonstrated to exert strong anticancer effects in various malignancies [8,9,10,11,21,29]. However, buformin was withdrawn from the markets in many countries and areas because of the risk of lactic acidosis. Currently, it is only prescribed to treat type 2 diabetes in several Eastern European and Latin American countries [17,30]. Recently, several studies have shown that buformin could be an effective anticancer agent, which presented greater lipophilicity and inhibition of mitochondrial complex I (a key regulator of ATP production) [7,31]. In a panel of endometrial cancer cell lines, buformin could lead to G1-phase arrest, enhance apoptosis, and decrease cellular adhesion and invasion [21]. Our western blot results demonstrated that these changes were caused by buformin-induced regulation of the AMPK/mTOR/S6 pathway [21].

In MMTV-erbB-2 transgenic mice which can be induced to develop breast cancer, treatment with buformin deactivated many signaling pathways including mTOR, ER, and β-catenin and eventually significantly impaired the stemness of breast cancer cells [19]. Consistently, in the present study, we proved that buformin can act as an AMPK activator and notably suppress the proliferation and invasion of osteosarcoma cells.

For most osteosarcoma patients, chemotherapy with platinum and paclitaxel is inevitable and potentially beneficial. As a suppressor of the abnormal cancer metabolism, buformin might be a powerful drug sensitizer; therefore, further research is warranted. In the present study, we examined the synergy between buformin and cisplatin. According to our *in vitro* data, a low dose of buformin strongly enhanced the cytotoxicity of cisplatin. Encouragingly, the same sensitizing effect was observed in primary cultured osteosarcoma tissues. These findings indicate that the synthetic use of buformin and routine chemotherapeutic drugs might be a novel choice in clinical settings.

Although buformin could significantly enhance cellular insulin sensitivity, it is prohibited in many markets because of the elevated risk of lactic acidosis [18,30]. However, some studies reported that buformin-induced lactic acidosis was only found in individuals with renal dysfunction, and it could be prevented by concomitant administration with 2-deoxyglucose or thiamine [32,33]. Therefore, the clinical trials evaluating the anticancer effects of buformin in osteosarcoma are necessary in the future.

In summary, we first demonstrated that buformin exhibits powerful suppressive effects on the proliferation and invasion of osteosarcoma, through activating the AMPK signaling pathway. Moreover, we proved that a low dose of buformin could notably increase tumor sensitivity to cisplatin. Collectively, these findings suggest buformin may serve as a novel agent for treating osteosarcoma.
